# Jet-Cooled Spectroscopy of Amino-Substituted Cinnamates

**DOI:** 10.1021/acs.jpca.6c04208

**Published:** 2026-08-01

**Authors:** Hugo Maurer, Wybren Jan Buma

**Affiliations:** † Van ’t Hoff Institute for Molecular Sciences, Faculty of Science, 1234 University of Amsterdam, Science Park 904, 1098 XH Amsterdam, The Netherlands; ‡ Radboud University, Institute for Molecules and Materials, FELIX Laboratory, Toernooiveld 7c, 6525 ED Nijmegen, The Netherlands

## Abstract

The photochemical and photophysical relaxation pathways of UV filters play a critical role in determining their stability and safety. Here, we present comprehensive gas-phase spectroscopic studies of cinnamate derivatives substituted on the phenyl group with an amino group in the *para* (M4AC), *meta* (M3AC), and *ortho* (M2AC) positions, while the influence of increasing the electron-donating character of the substituent has been investigated by studies on the *para*-dimethylamino derivative (MeM4AC). Using Resonance Enhanced Multiphoton Ionization (REMPI), UV–UV depletion, pump–probe ion yield measurements, and photoelectron velocity-map imaging (VMI), complemented by electronic structure calculations, we characterize their electronically excited-state structure and dynamics. Compared to previously studied hydroxy- and methoxy-substituted cinnamates, all four derivatives exhibit substantial changes in electronic structure and excited-state behavior. Amino substitution is found to lead to pronounced red shifts of the S_1_ absorption band and to modify the ordering of the low-lying excited states. Time-resolved measurements show nanosecond excited-state lifetimes that are dominated by radiative and nonradiative relaxation from the S_1_ state to the ground state, while population transfer to long-lived ^1^nπ* states -that was previously shown to promote intersystem crossing to the triplet manifold in hydroxy- and methoxy-substituted cinnamates- is strongly suppressed. These results demonstrate that amino substitution has a profound influence on the properties of cinnamate-based UV filters, providing a new molecular design approach to improve UV-absorbing molecular materials.

## Graphical abstract

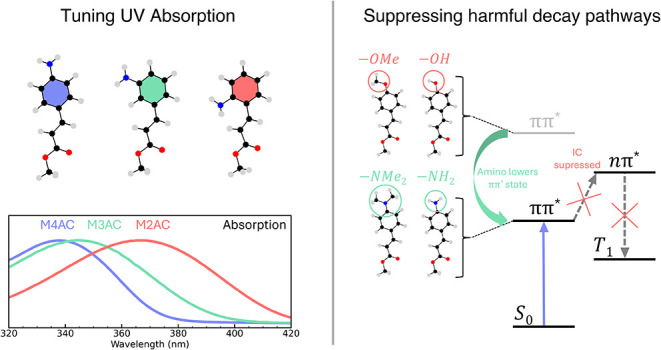

## Introduction

Global warming, caused by human activities,^1^ will go down in history as the greatest challenge of the 21st century. Among the multitudes of new problems that climate change brings, the increase in the rate of skin cancer in the global population is of major concern.^2–4^ The main cause of skin-induced cancer is exposure to ultraviolet (UV) radiation. As the temperature globally rises and the stratospheric ozone layer depletes, humans are more exposed to UV radiation. This radiation can be divided into three categories of energies inversely proportional to their wavelength: UVA (320–400 nm), UVB (290–320 nm), and UVC (100–290 nm). While UVC radiation is completely absorbed by the ozone layer in the stratosphere, both UVB and UVA reach the Earth’s surface. UVB radiation can be directly absorbed by DNA bases^5^ whereas UVA radiation primarily induces DNA damage indirectly through the formation of photooxidation products.^6^

Nature found many solutions to protect itself from these harmful radiations, one of them being the use of UV-absorbing filters. The strategy behind these filters is that they absorb UV radiation and dissipate its energy into harmless thermal energy. Such photoprotectors are naturally found in various organisms, like mycosporines-like amino acids (MAAs) found in cyanobacteria and algae,^7^ or melanin found in the human skin.^8^ Commercially available chemical sunscreens use organic UV-filters to protect the user, such as ethylhexyl-4-methoxycinnamate (EHMC), a widely used cinnamate-based UVB absorber. Although EHMC efficiently converts absorbed UV radiation into thermal energy, it has been shown that photoexcitation can populate a long-lived electronic excited state,^9,10^ which may lead to the generation of reactive oxygen species upon prolonged irradiation.^11,12^ At the same time, there are increasing concerns regarding potential health and environmental effects of many commonly used UV filters.^13,14^ These concerns led the U.S. Food and Drug Administration (FDA) in 2021 to revise the GRASE status of a large number of chemical sunscreens.^15^ As a consequence, there is a strong demand to develop UV filters that are safer, more efficient, and environmentally harmless.

A broad range of substituted cinnamates are found in nature, displaying diverse substitution patterns while sharing a common molecular backbone.^16–18^ These naturally occurring compounds have served as templates for the development of synthetic UV filters in a variety of applications.^19–21^ The strategy of the present work is to exploit the cinnamate scaffold and tailor its substitution pattern in order to control the excited-state energy dissipation pathways. Previous studies in our group have focused on cinnamate derivatives substituted with oxygenated functionalities such as hydroxy (OH) or methoxy (OMe) groups.^20,22–25^ These substitutions were shown to significantly influence the excited-state dynamics. However, the dissipation of the absorbed energy was still not optimal as intersystem crossing (ISC) to the triplet manifold remained one of the active decay pathways albeit that its contribution was found to depend sensitively on the details of the substitution pattern.

In the present studies we therefore investigate the spectroscopic properties of methylaminocinnamate derivatives substituted with an amino (NH_2_) group in the *para* (M4AC), *meta* (M3AC) and *ortho* (M2AC) position of the phenyl ring (Figure 1). Due to its strong electron donating character, the amino group is expected to modulate the electron density of the conjugated π system, thereby influencing the relative energies and accessibility of the ^1^ππ* and ^1^nπ* excited states as well as the energies of triplet excited states, which could be of importance for ISC rates. Substitution in the *ortho* position may moreover induce nonplanarity, thereby partially decoupling the conjugated π system and altering the charge-transfer character of excited states. Furthermore, in kynurenines, naturally occurring UV-filters found in the human eye, the *ortho*-substituted amino group plays a crucial role in the dissipation of absorbed UV energy through excited-state intramolecular proton transfer (ESIPT).^26,27^ Although such a mechanism is not operational in the cinnamate derivatives studied here, M2AC is an interesting compound to explore in the context of understanding the effects of *ortho*-amino substitution. Additionally, to further examine substitution with strongly electron-donating moieties, we also investigate methyl *para*-dimethylamino cinnamate (MeM4AC), in which the substituent is an even stronger electron donor than NH_2_.

**Figure 1:**
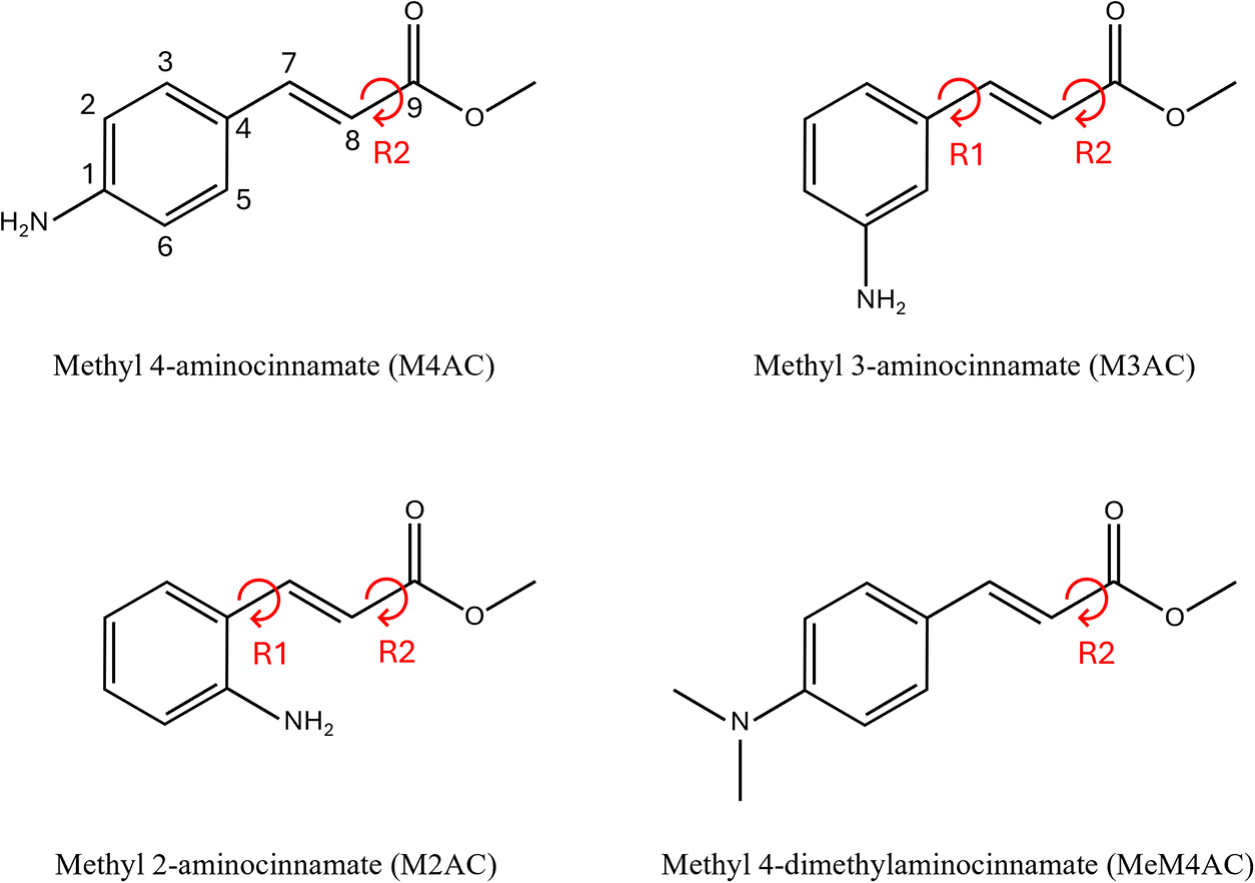
Molecular structure of the four molecules of interest in this study, M4AC, M3AC, M2AC and MeM4AC. The relevant rotatable bonds are indicated by red arrows: Rotator 1 (R1) corresponds to rotation around the phenyl–vinyl bond, giving rise to *syn* and *anti* conformers, while rotator 2 (R2) corresponds to rotation around the ester bond, leading to s-*cis* and s-*trans* conformations. The carbon backbone atom numbering used throughout this work is shown for M4AC.

We present comprehensive gas-phase spectroscopic studies of jet-cooled M4AC, M3AC, M2AC and MeM4AC in which Resonance Enhanced MultiPhoton Ionization (REMPI) spectroscopy is employed to record vibrationally resolved excitation spectra. Excited-state lifetimes have been determined using pump–probe ion-yield spectroscopy, and have been complemented by low- and high-kinetic-energy velocity map imaging (VMI) measurements of the photoelectrons produced upon ionization. In combination with quantum chemical calculations, our results show that the electron-donating amino substituent drastically alters the excited-state ordering and dynamics, and their dependence on the position on the phenyl ring where substitution takes place. In particular, the amino group stabilizes the lowest ^1^ππ* excited state, thereby strongly suppressing population transfer to the ^1^nπ* state and subsequent ISC to a detrimental long-lived triplet ^3^ππ* state. These findings point toward a molecular design strategy in which strong electron-donating substituents are used to tailor the relaxation pathways of electronically excited states of UV-absorbing molecules.

## Methods

### Experimental Details

The spectrometer and methods have previously been described in detail.^22,28^ In the following we will therefore only provide a brief summary and indicate the modifications relevant to the present studies. The molecules studied in this article (methyl 4-aminocinnamate (M4AC), methyl 3-aminocinnamate (M3AC) and methyl 2-aminocinnamate (M2AC)) were purchased at abcr GmbH. Methyl 4-dimethylaminocinnamate (MeM4AC) was synthesized in-house via a Wittig reaction between 4-dimethylamino-benzaldehyde and (2-hydroxy-2-methoxyvinyl) triphenylphosphonium hydroxide. Their structures can be seen in Figure 1. A stainless steel oven containing a glass sample holder was heated to ∼140 °C to achieve sufficient vapor pressure. A cold molecular beam with rotational temperatures of 2–10 K and vibrational temperatures around 20 K was then generated by mixing this vapor with neon gas at a backing pressure of 2 bar, and expanding it into a vacuum using a pulsed valve. After passing a 1 mm skimmer the beam entered the ion source region equipped with a three-plate electrostatic lens similar to the one designed by Parker and Eppink^29^ that allowed for either kinetic-energy-resolved electron detection using electron Velocity Map Imaging (e-VMI), or time-of-flight (TOF) based mass-resolved ion detection.

Excitation spectra have been recorded with either one-color (1 + 1) Resonance Enhanced Two-Photon Ionization (R2PI) using a Nd:YAG pumped dye laser with a resolution of ∼0.07 cm^–1^, or two-color (1 + 1′) R2PI using the aforementioned dye laser system for excitation and an ArF excimer laser (193 nm) for ionization. Time-resolved decay curves have been obtained by varying the delay between excitation and ionization laser pulses in steps of 1 ns. For UV–UV depletion studies, two Nd:YAG pumped dye lasers have been employed. In all cases laser beams were linearly polarized parallel to the detector plane. M4AC, M3AC, M2AC and MeM4AC have been excited using DCM, LDS 698, LDS 722 and LDS 698, respectively.

Photoelectron images are the sum of the photoelectrons generated from typically tens of thousands laser shots, captured by our camera (Andor Zyla sCMOS) for each time delay and then compiled into a single image. A photoelectron background was subtracted from the images by setting the ionization laser pulse earlier (−100 μs) relative to the excitation laser pulse, removing the noise from background gases, the excimer laser and potential (1 + 1) signals. The VMI images were circularized to correct for deformation and geometrically centered with an adapted version of the method from Gascooke et al.^30^ These images were then subjected to an inverse Abel transform using the rBASEX algorithm^31^ to reconstruct the central slice of the original 3D Newton sphere, allowing for an appropriate velocity and radial analysis. The radial axis of the integrated Abel inversed images were scaled into energy using a calibration of our experimental setup on well-known multiphoton transitions of xenon atoms. The intensity axis of the same plots were corrected by a Jacobian. All data were processed and plotted using Python 3.10 ^32^ and Numpy 1.26.4.^33^ The VMI images have been analyzed using Scipy 1.13.1 ^34^ as well as PyAbel 0.9.0.^35^

### Computational Details

For each of the rotamers associated with the single-bond rotations of the systems depicted in Figure 1, geometry optimization of the S_0_, D_0_, and T_1_ states has been performed using (Unrestricted) Density Functional ((U)­DFT) at the ωB97XD/cc-pVDZ level,^36,37^ while Time Dependent DFT (TD-DFT) has been employed to optimize the geometries of electronically excited states of the neutral and cation, and to determine harmonic force fields. Adiabatic ionization energies have been determined from the energies of the pertaining electronic states of the neutral and cation at their respective equilibrium geometries. Vertical ionization energies have been determined from (TD)-UDFT calculations on the ionic states at the equilibrium geometries of the electronic state of interest of the neutral molecule. It should be noticed that quantum chemical calculations show that T_1_ can adopt two equilibrium geometries corresponding to quasi-planar and twisted structures, as has also been observed for other cinnamates.^16,22^ However, their energy difference is smaller than the S_1_–T_1_ energy gap. Internal conversion from S_1_ to T_1_ will thus populate highly excited vibrational levels of T_1_ that reflect an effective planarity of the molecule. We therefore take for the kinetic energies associated with ionization of T_1_ the ones calculated for the quasi-planar structure. All calculations have been performed using the Gaussian16, Rev. C.02 suite of programs.^38^

## Results and Discussion

### Methyl 4-Aminocinnamate

Figure 2a shows the two-color (1 + 1′) R2PI excitation spectrum of M4AC. A similar spectrum is obtained using one-color (1 + 1) R2PI, indicating that at these excitation energies two photons are sufficient to reach the ionization continuum. However, on the basis of the higher intensities of the bands in the (1 + 1′) spectrum we conclude that the cross section for ionization with 193 nm is larger than in the one-color case, most likely due to a more favorable Franck–Condon overlap. Previous studies on coumarates^20,25^ and *a priori* considerations of the conformational heterogeneity of the molecule lead us to expect that at room temperature the molecule will be present in two conformations, s-*cis* and s-*trans*, and thus that the spectrum displayed in Figure 2a is actually composed of multiple contributions. Such an expectation is indeed confirmed by UV depletion experiments, for which spectra are given in Figure 2b. In these depletion experiments, the intensity of the depletion laser was kept high to ensure a proper assignment of the conformer responsible for a particular band. Under such conditions extensive saturation of transitions will have occurred, and the depletion depths therefore will not reflect the intrinsic Franck–Condon factors of the transitions. What for the present purpose is more important, however, is that these experiments clearly show two spectra with 0–0 transitions at 30698 cm^–1^ and 30898 cm^–1^ (red and blue traces in Figure 2b). Further confirmation of the conclusion that the R2PI spectrum has contributions from two conformations is obtained from our quantum chemical calculations that predict (i) that the s-*cis* conformation is the lowest-energy conformation with a room-temperature Gibbs energy difference of 324 cm^–1^ with respect to the s-*trans* conformation, and (ii) that the S_0_ → S_1_ 0–0 transition for s-*cis* occurs 170 cm^–1^ lower in energy than for s-*trans*. We thus assign the spectrum with the 30698 cm^–1^ 0–0 transition to the excitation spectrum of s-*cis*, and the spectrum starting at 30898 cm^–1^ to s-*trans*. In previous studies^25,39^ bands were often observed in the molecular ion mass channel that originated from clusters with water. Such was not the case in the present experiments, all bands observed in the R2PI excitation spectrum can be assigned to either the s-*cis* or s-*trans* conformer.

**Figure 2:**
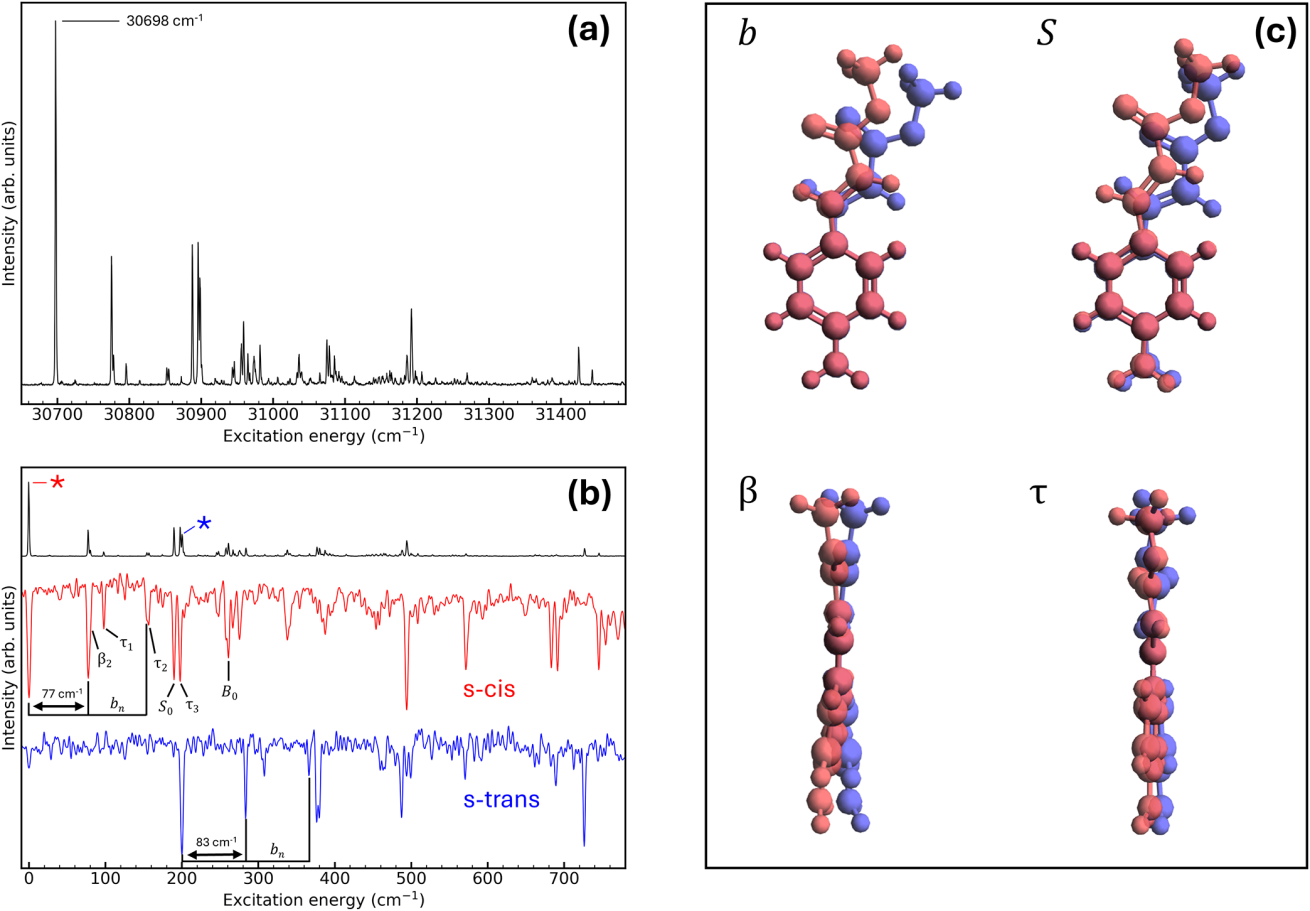
(a) (1 + 1′) R2PI excitation spectrum of M4AC. (b) UV–UV depletion spectra of the s-*cis* (red) and s-*trans* (blue) conformers of M4AC probed at 30698 cm^–1^ and 30898 cm^–1^, respectively. The depletion spectra have been processed with a weak low-pass filter and their slope have been corrected for ease of visualization. The identified vibrational modes have been annotated in the spectra. (c) Molecular structure of M4AC displaying some of the more active low-frequency vibrations: *b*, C_4_–C_7_=C_8_ in-plane bending; *S*, C_7_=C_8_–C_9_ in-plane scissoring; β, butterfly; τ, out-of-plane twisting.

Studies on methyl *para*-methoxy methylcinnamate (*p*-MMC)^40^ reported a lowest-energy band at 32328 cm^–1^ that was attributed to the S_0_ → S_1_ 0–0 transition of the *syn/s-cis* conformation. The red shift of 1630 cm^–1^ of M4AC relative to *p*-MMC can be attributed to the strong electron-donating character of the amino substituent. This group increases the energy of the HOMO more significantly than that of the LUMO, owing to the distribution of electron density in the *para* position, thereby reducing the HOMO–LUMO gap. For *p*-MMC, the *syn/s-cis* and *syn/s-trans* conformers have 0–0 transition energies at 32328 cm^–1^ and 32587 cm^–1^, respectively. This splitting of 259 cm^–1^ is comparable to that observed here for M4AC (200 cm^–1^), consistent with the fact that the local structure around the rotatable bond R2 remains essentially unchanged.

Analysis of the Franck–Condon activity of the M4AC s-*cis* conformer leads to the conclusion that reasonably strong in-plane activity is observed of the in-plane C_4_–C_7_=C_8_ bending mode at 77 cm^–1^ (mode *b*), the in-plane C_7_=C_8_–C_9_ scissoring mode at 190 cm^–1^ (mode *S*) and another C_3_–C_4_–C_7_ in-plane bending mode at 260 cm^–1^ (mode *B*). Activity of the out-of-plane butterfly mode at 26 cm^–1^ (mode β) and twisting mode at 52 cm^–1^ (mode τ) is observed, but only in the form of overtones or combination bands. These bands have been annotated on the depletion spectra shown in Figure 2b, with the atomic displacements of some of the modes involved displayed in Figure 2c. For the s-*trans* conformer, Franck–Condon activity of the same vibrational modes are observed, with once again the in-plane bending mode *b* at 83 cm^–1^ being most prominently visible.

M4AC formally does not have elements of symmetry because of the nonplanar amino group. The observation that Franck–Condon activity nevertheless appears to be restricted to modes that loosely speaking involve “in-plane” displacements as well as to overtones and combination bands of “out-of-plane” modes suggests that excitation to S_1_ is not accompanied by significant displacements along out-of-plane coordinates, i.e., in both S_0_ and S_1_ quasi-planarity is retained. An alternative explanation is that the barrier associated with inversion of the amino group is sufficiently low in ground and electronically excited states such that the zero-point energy level of this mode is above this barrier. As a result, the molecule would spectroscopically appear to be planar. Further insight into the effective planarity of M4AC could be provided by the (non)­observation of a tunneling splitting in rotational spectroscopy studies.

Previous studies^20–22^ on sinapate derivatives reported REMPI excitation and UV–UV depletion spectra for these systems. For molecules exhibiting relatively simple and well-resolved spectra such as sinapic acid (SA) and methyl sinapate (MS), the vibrational assignments proposed in those works are similar to the assignments made here for M4AC. A notable difference, however, is observed in the progression of the in-plane bending mode *b*. The sinapate derivatives display longer progressions, with up to three or more observable overtones, whereas in M4AC only two overtones are detected. This reduced progression suggests a smaller displacement along this normal coordinate in the excited state of M4AC, and thus a more limited geometry change upon excitation compared to these previously studied systems.

A final point of attention concerns the observation that no significant vibrational activity is detected beyond approximately 800 cm^–1^ above the 0–0 transition. Franck–Condon vibrational overlap calculations predict in contrast that from a vibrational overlap point of view such activity should still be present. Similar observations have been made in molecular beam studies on other substituted cinnamates.^21,25,39^ In previous studies^25^ we tentatively concluded that this might indicate an additional decay channel opening up in the electronically excited state. However, with the realization that around such energies the state density is on the order of 10^2^/cm^–1^ [Fn fn1] it is more likely that this apparent cutoff should be attributed to a redistribution of the transition moment of the active Franck–Condon mode over nearby vibronic levels, leading to spectral broadening and a loss of resolvable structure.

Figure 3a shows the nanosecond pump–probe normalized ion yield of M4AC probed at the 0–0 transition of the s-*cis* conformer at 30698 cm^–1^. The signal was best fitted with a biexponential decay convoluted with the instrument response function (IRF) of our instrument. The IRF was measured experimentally and is adequately described by a Gaussian function with a full width half-maximum of 7 ns. From such fits we find decay times of 4.8 ± 0.1 ns and 45 ± 11 ns, with the short-lived component accounting for 97.5% of the signal amplitude and the long-lived component contributing 2.5%. Similar to our previous studies,^9,25,39^ we attribute the short lifetime to a combination of fast radiative and nonradiative decay pathways from S_1_ to S_0_ as well as intersystem crossing of S_1_ to the triplet manifold, while the long lifetime is associated with the nonradiative decay from T_1_ to the ground state. Pump–probe measurements performed at higher excitation energies and at bands associated with the s-*trans* conformer were likewise well described by a biexponential model. The fitted lifetimes remain essentially unchanged, although the relative amplitude of the long-lived component increases gradually with excitation energy, reaching approximately 5% at 31194 cm^–1^.

**Figure 3:**
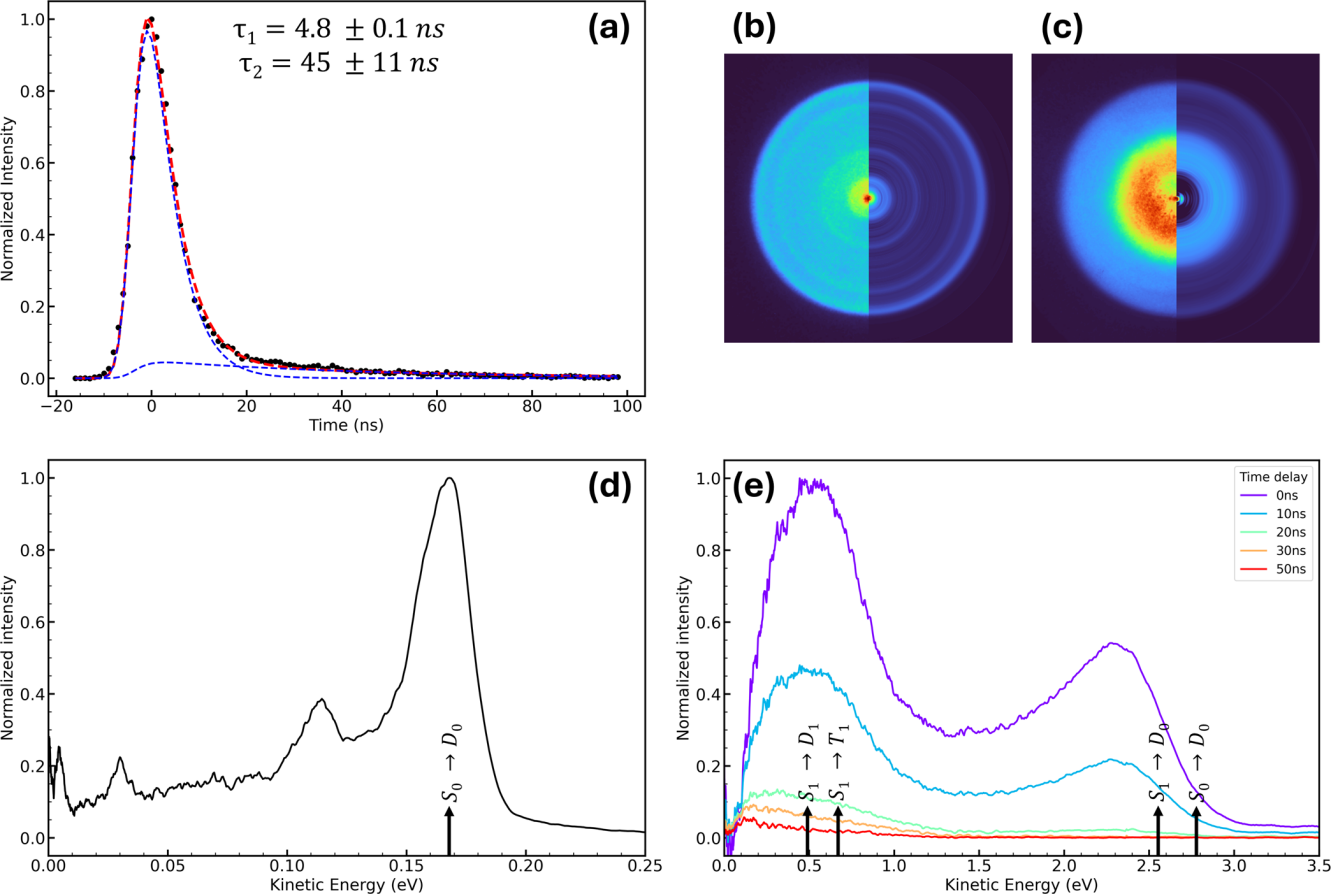
(a) Pump–probe ion yield of M4AC probed at 30698 cm^–1^. The black dots represent the data, the red dashed line is the full fit of the data, while the blue dashed lines depict the individual exponential decay components. One-color (b) and two-color (c) PE velocity-map images (VMIs) of M4AC; the left and right halves show the raw and Abel-inverted images, respectively. (d) (1 + 1) PE spectrum of M4AC probed at 30698 cm^–1^. (e) (1 + 1′) PE spectra of M4AC excited at 30698 cm^–1^ and ionized at 193 nm recorded at different time delays between excitation and ionization laser pulses.

Importantly, these decay dynamics are fundamentally different from those of *p*-MMC, the methoxy counterpart of M4AC,^9^ as well as other hydroxy- and methoxy-substituted cinnamates including para-coumaric acid, methyl 4-hydroxycinnamate, ferulic acid, and 2-ethyl-hexyl-4-methoxycinnamate (EHMC).^28,40,42,43^ In that case a single exponential decay was observed with a decay time of 24 ± 0.2 ns that was explained by a fast internal conversion from the excited ^1^ππ* state to a lower-lying ^1^nπ* state, followed by efficient intersystem crossing to the triplet manifold because of the El-Sayed rule,^44^ from which the T_1_ state decayed on the observed ns time scale. We thus conclude that introducing a stronger electron donating substituent at the *para* position impedes such an efficient intersystem crossing pathway, the most logical explanation being that the ^1^nπ* state is not accessible anymore in M4AC. Such an explanation is confirmed by our calculations (see Table 1) which show that for vertical excitation the ^1^nπ* state is S_3_. Upon geometry relaxation, this state becomes S_2_ but thus not lower in energy than the lowest ^1^ππ* state as is the case for *p*-MMC.

**Table 1:** Vertical and Adiabatic *ω*B97XD/cc-pVDZ Electronic Excitation Energies (eV) of the Lower-Lying Electronically Excited Singlet States of the Lowest-Energy Conformers of M4AC (s-*cis*), M3AC (anti/s-*cis*), M2AC (syn/s-*cis*) and MeM4AC (s-*cis*) with Oscillator Strength Given in Parentheses

	M4AC		M3AC		M2AC		MeM4AC	
	Vertical	Adiabatic	Vertical	Adiabatic	Vertical	Adiabatic	Vertical	Adiabatic
1^1^ππ*	4.391	4.110	4.226	3.897	4.134	3.605	4.187	3.960
	(0.78)	(0.80)	(0.13)	(0.11)	(0.24)	(0.16)	(0.91)	(0.94)
2^1^ππ*	4.799	4.626	4.825	4.442	4.960	^a^	4.659	4.492
	(0.09)	(0.13)	(0.56)	(0.55)	(0.20)		(0.08)	(0.10)
1^1^nπ*	5.010	4.358	4.936	4.290	5.007	^a^	5.024^b^	4.380^b^
	(2 × 10^–4^)	(0.0000)	(7 × 10^–4^)	(0.0000)	(0.06)		(2 × 10^–4^)	(0.0000)

a Geometry optimization leads to root switching with the 1^1^ππ* state.

b Obtained under *C*_s_ symmetry constraint. Without symmetry, S_3_ converges to the 1^1^ππ* state.

Figure 3d shows the photoelectron (PE) spectrum obtained after one-color excitation and ionization at 30698 cm^–1^ of the s-*cis* conformer of M4AC with the S_0_ → D_0_ transition annotated on the spectrum. Under such conditions the total absorbed photon energy is slightly larger than the adiabatic ionization energy of D_0_, leading to photoelectrons with very low kinetic energies. As such, they allow for an accurate determination of this adiabatic ionization energy, which is found to be 7.444 ± 0.005 eV for the s-*cis* conformer of M4AC. Similarly, a one-color PE of the s-*trans* conformer at 30898 cm^–1^ was recorded, and its ionization energy is found to be at 7.466 ± 0.005 eV. Compared to *p*-MMC for which an ionization energy of 8.078 eV has been determined,^42,45^ this implies, loosely speaking, that replacing the methoxy group by an amino group raises the energy of the HOMO by 0.634 eV. Interestingly, Figure 3b shows that under the employed conditions vibrational resolution in the VMI image is to a large extent still retained. Closer inspection of the 0.168 eV band reveals that it actually is composed of a progression of bands with a spacing of ∼0.010 meV (∼81 cm^–1^) associated with the in-plane C_4_–C_7_=C_8_ bending mode in the ion. The band at 0.113 eV (∼440 cm^–1^) can be assigned to another in-plane bending mode of the ion.

The VMI image obtained at the same excitation energy but using an ArF excimer laser (193 nm) for ionization is displayed in Figure 3c. The integrated PE spectrum is shown in Figure 3e together with the energies predicted for vertical ionization of S_1_ and T_1_ to different ionic states. The S_0_ → D_0_ arrow indicates the PE energy associated with adiabatic ionization of the ground state and thus the maximum kinetic energy photoelectrons might have under the employed conditions. Importantly, for these predictions the experimentally determined adiabatic ionization energy of D_0_ has been used in combination with adiabatic and vertical ionization energies to the electronically excited states of the ion calculated using the (TD)-UDFT excitation energies of these states. At zero time delay between excitation and ionization, the PE spectrum is observed to have two distinct bands. The higher kinetic energy band at 2.3 eV is in excellent agreement with electrons originating from a S_1_ → D_0_ ionization pathway for which a kinetic energy of ∼2.5 eV is predicted. The lower kinetic energy band at 0.6 eV could in principle have contributions from a S_1_ → D_1_ and a T_1_ → D_0_ ionization pathway but based on the time dependence of this band (*vide infra*) it can be concluded that it is dominated by the S_1_ → D_1_ pathway.

Upon increasing the time delay between excitation and ionization lasers up to 50 ns, both bands are observed to decay, but it is also observed that that their relative intensities change. In fact, for time delays longer than ∼20 ns a band remains at ∼0.5 eV, slightly shifted from the time zero band at ∼0.6 eV. We thus conclude that S_1_ dominantly decays via radiative and nonradiative pathways to the ground state, but that there still remains a small contribution from a triplet-mediated pathway, in agreement with what was concluded from the time-dependent pump–probe ion yield curves.

### Methyl 3-Aminocinnamate

The (1 + 1′) R2PI excitation spectrum of M3AC is shown in Figure 4a. A one-color (1 + 1) spectrum could not be recorded, indicating that two photons at these excitation energies are insufficient to reach the ionization continuum. The lowest-energy transition of M3AC is found at 28505 cm^–1^, corresponding to a red shift of more than 2000 cm^–1^ relative to what is observed for M4AC. In addition, M3AC displays significantly more vibrational structure, with resolved activity extending up to approximately 1300 cm^–1^ above the origin. UV–UV depletion experiments reveal the presence of three conformers in the molecular beam with origin bands at 28505, 28527, and 28755 cm^–1^ with conformation-specific spectra shown in Figure 4b. Based on quantum chemical calculations that predict the s-*cis* conformers to be more stable than the s-*trans* conformers as well as predicted S_0_ → S_1_ 0–0 transition energies (Table S1) we assign the depletion spectra associated with these origin bands to the *anti*/s-*cis*, *syn*/s-*cis*, and *syn*/s-*trans* conformers, respectively. Although based on the energies reported in Table S1 one would expect the *anti*/s-*trans* conformer to be present as well, it was not observed, indicating that its relative Gibbs energy is quite probably higher than reported in Table S1. Comparison with *meta*-methoxycinnamate (*m*-MMC), for which the lowest-energy 0–0 transition was reported at 31120 cm^–1^,^40^ shows that the red shift upon going from the methoxy to the amino substituent is larger than what is observed for *para* substitution. This difference can be traced back to the fact that for the *meta* substitution the HOMO is raised in energy, but the LUMO is not affected because it has a node at the *meta* position.

**Figure 4:**
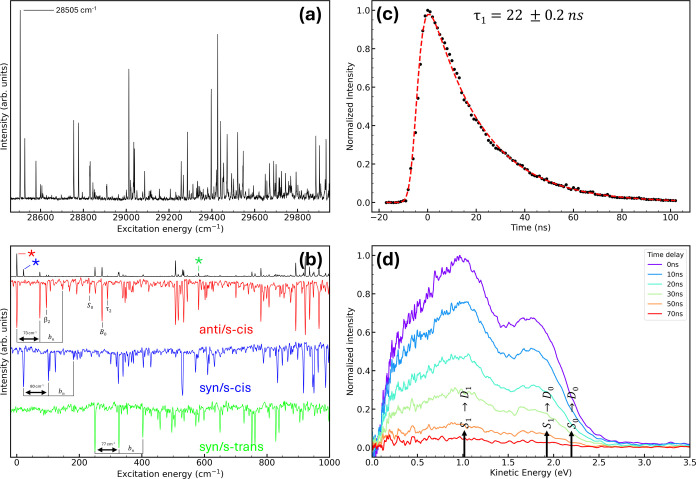
(a) (1 + 1′) R2PI excitation spectrum of M3AC. (b) UV–UV depletion spectra of the *anti*/s-*cis* (red), *syn*/s-*cis* (blue) and *syn*/s-*trans* (green) conformers probed at 28505 cm^–1^, 28527 cm^–1^ and 29257 cm^–1^, respectively. The depletion spectra have been processed with a weak low-pass filter and their slope has been corrected for ease of visualization. (c) Pump–probe ion yield of M3AC probed at 28505 cm^–1^. The black dots represent the data while the red dashed line is the full fit of the data. (d) (1 + 1′) PE spectra of M3AC excited at 28505 cm^–1^ and ionized at 193 nm, recorded at different time delays between excitation and ionization laser pulses.

Focusing on the *anti*/s-*cis* conformer, the dominant vibrational features can be assigned to modes that are similarly active in M4AC. The more intense bands arise from in-plane backbone motions, including a low-frequency bending mode at 73 cm^–1^ (mode *b*) around C_4_–C_7_=C_8_, another bending mode at 272 cm^–1^ (mode *B*) around C_3_–C_4_–C_7_, and a scissoring mode at 251 cm^–1^ (mode *S*) around C_7_=C_8_–C_9_. Weaker contributions originate from out-of-plane motions, like the second overtones of a butterfly mode at 31 cm^–1^ (mode β) and a twisting mode at 97 cm^–1^ (mode τ). Similar vibrational assignments can be made for the *syn*/s-*cis*, and *syn*/s-*trans* conformers. As in M4AC, the vibrational progressions are relatively short, with only a few observable overtones. The overall conclusion that can be drawn is that similar to M4AC, the molecule is not accompanied by significant displacements along out-of-plane coordinates, i.e., in both S_0_ and S_1_ quasi-planarity is retained. Quantum chemical calculations find indeed an sp^2^ hybridization of the amino group in S_1_. In the ground state, however, the group is nonplanar with a sum of nitrogen bond angles of ∼340°. We therefore conclude that in the ground state the barrier associated with inversion of the amino group is lower than the zero-point energy level of this mode making the molecule effectively appear to be planar.

Figure 4c displays the ion yield time trace obtained by pump–probe spectroscopy after excitation of the 0–0 transition of the *anti*/s-*cis* conformer at 28505 cm^–1^. The decay is well described by a monoexponential function, giving a lifetime of 22 ± 0.2 ns. This single lifetime is attributed to combined radiative and nonradiative relaxation pathways from S_1_ to the ground state. In contrast to M4AC for which a biexponential decay model was required to describe the dynamics, no additional slow component is observed here, indicating that the secondary decay channel is effectively closed. Measurements performed at higher excitation energies also required a fit with a monoexponential model with lifetimes that remain essentially unchanged. The absence of the slow decay component can be explained by the relative energies of the lower electronically excited states. The calculated adiabatic energy of the ^1^nπ* state (4.290 eV) lies above the vertical excitation energy of the ^1^ππ* state (4.226 eV) (Table 1). Efficient coupling between these states is therefore unlikely, preventing population transfer from the S_1_ (^1^ππ*) state to the ^1^nπ* state through internal conversion. A similar behavior was reported for *m*-MMC for which as well a single lifetime of approximately 10 ns was observed.^40^ The calculations reported in that study showed that internal conversion from the ^1^ππ* state to the ^1^nπ* state is inefficient because of the substantial energy barrier separating the two states. Relaxation therefore predominantly occurs through radiative decay from the S_1_ state leading to the relatively long excited-state lifetime.

Two-color PE spectra of M3AC recorded at different ionization pulse delays are shown in Figure 4d. In this case a one-color VMI spectrum could not be obtained since the energy of two excitation photons is not enough to reach the ionization continuum. It was thus not possible to calibrate the computationally predicted PE energies for various ionization pathways using an experimental value for the S_0_ → D_0_ adiabatic ionization energy. We have therefore taken in this case the onset of ionization observed for photoelectrons at 2.2 eV as the S_0_ → D_0_ adiabatic ionization energy. At zero delay, two principal features are observed. The higher kinetic energy band around 1.8 eV is assigned to the S_1_ → D_0_ ionization pathway while the lower kinetic energy one around 1.0 eV is attributed to ionization of S_1_ to D_1_. Both bands decay uniformly with increasing pump–probe delay, demonstrating that the photoelectrons originate from the same electronic state. Important to notice and in contrast to M4AC is that no residual low-kinetic-energy signal is observed at longer time delays. This absence of a delayed component indicates that ionization from T_1_ does not contribute to the spectra as was also concluded from the pump–probe experiments. Both pump–probe as well as the PE spectra thus demonstrate that the lack of a slow decay component should be attributed to negligible intersystem crossing to the triplet manifold, most probably as the result of the absence of internal conversion to the ^1^nπ* state.

### Methyl 2-Aminocinnamate

Compared to the two molecules discussed previously, the R2PI excitation spectrum of M2AC, which is shown in Figure 5a, is drastically different, with extensive vibrational activity indicating that a significant change in geometry occurs upon excitation. A priori considerations lead us to expect that four low-energy conformers are present as is indeed confirmed by quantum chemical calculations that predict the *syn/s-cis* conformation to be lowest in energy. UV–UV depletion measurements at 27428 cm^–1^ (red) and 27475 cm^–1^ (blue) (Figure 5b) reveal in contrast only the presence of two distinct species in the molecular beam under the present experimental conditions with S_0_ → S_1_ 0–0 transitions at 27239 cm^–1^ for the red species and 27286 cm^–1^ for the blue species. For both species, a dominant long vibrational progression are observed with a frequency of 94 cm^–1^. The large number of observed overtones extending up to about six - seven overtones indicates that it is in particular along this normal mode that a major geometry change occurs upon excitation. Due to the complexity of the vibrational activity in the excitation spectrum of M2AC, a full assignment and detailed interpretation of these spectra will be subject of a future study.

**Figure 5:**
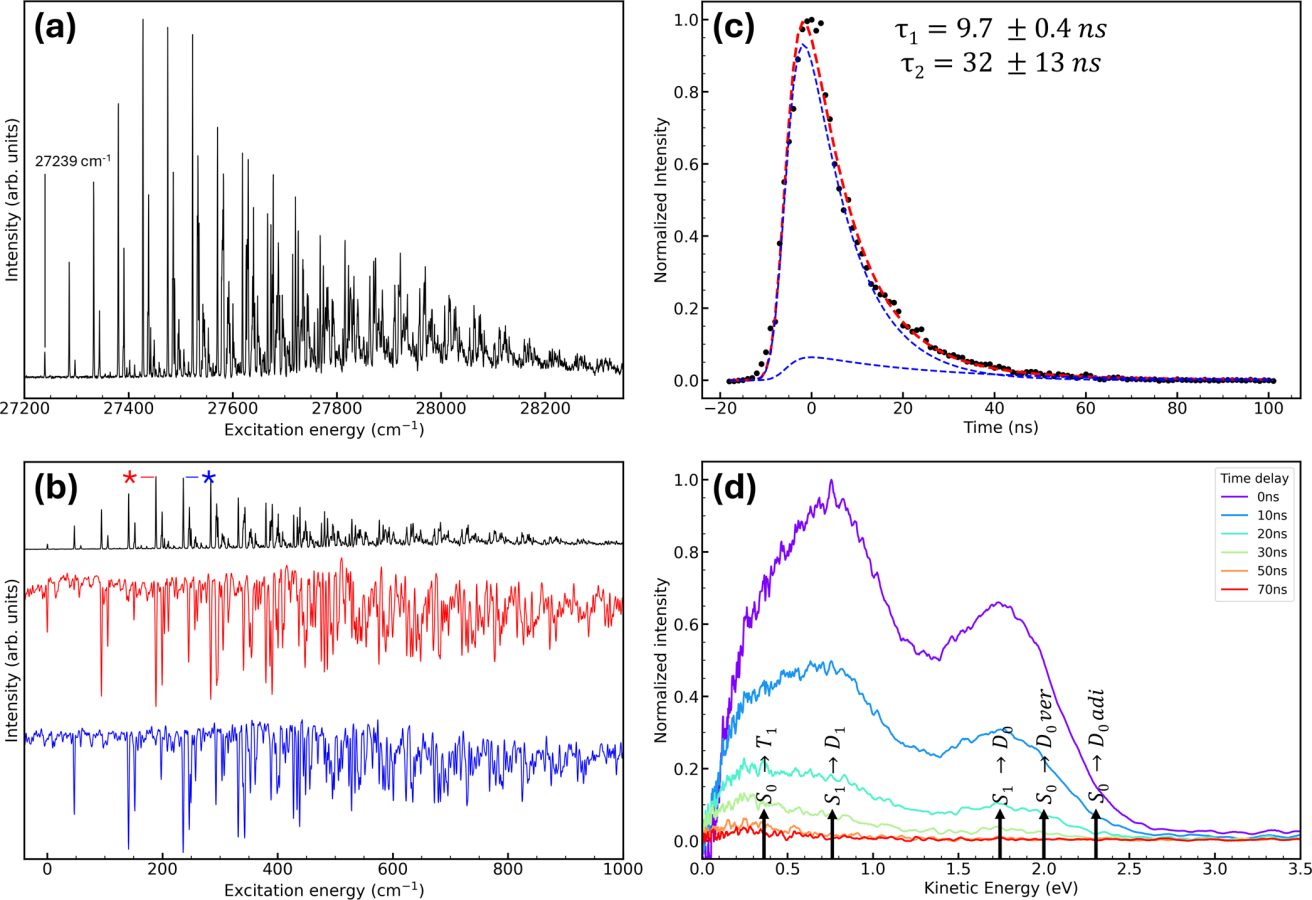
(a) (1 + 1′) R2PI excitation spectrum of M2AC. (b) UV–UV depletion spectra (red and blue) of two species of M2AC found in the molecular beam probed at 27428 cm^–1^ and 27475 cm^–1^, respectively. The depletion spectra have been processed with a weak low-pass filter and their slope has been corrected for ease of visualization. (c) Pump–probe ion yield of M2AC probed at 27428 cm^–1^. The black dots represent the data, the red dashed line is the full fit of the data while the blue dashed lines depict the individual exponential decay components. (d) (1 + 1′) PE spectra of M2AC excited at 27428 cm^–1^ and ionized at 193 nm, recorded at different time delays between excitation and ionization laser pulses.

Also in comparison to its methoxy-substituted counterpart (methyl *ortho*-methoxycinnamate (*o*-MMC)) studied previously by Miyazaki et al.^40^ striking differences are observed. The excitation spectrum of M2AC is shifted to the red by more than 3000 cm^–1^ compared to *o*-MMC, indicating a substantial reduction of the HOMO–LUMO gap for M2AC. In terms of vibrational activity, the two molecules differ as well. M2AC shows extensive vibrational structure with long progressions, pointing toward significant geometric distortions most likely involving out-of-plane displacements in the excited state. The LIF excitation spectrum of *o*-MMC reported in ref,^40^ on the other hand, shows more activity than the *para* and *meta* isomers, but appears to be somewhat saturated inhibiting a reliable comparison. It nevertheless lacks the low-frequency mode progression clearly observed for M2AC. These observations suggest that substitution of an amino group in the *ortho* position of methyl cinnamate has a larger impact on the structure of methyl cinnamate upon excitation than a methoxy group.

Pump–probe ion-yield spectroscopy of M2AC leads to decay traces as shown in Figure 5c for excitation at 27428 cm^–1^. The signal is well described by a biexponential decay, yielding lifetimes of 9.7 ± 0.4 ns and 32 ± 13 ns. The short-lived component accounts for approximately 95.0% of the total signal, while the long-lived component contributes about 5.0%. For *o*-MMC a single lifetime of ∼10 ns was reported^40^ that was assigned as the radiative lifetime of the S_1_(^1^ππ*) state of this compound. In contrast, the lifetimes and relative amplitudes observed for M2AC are very similar to those found for M4AC, suggesting similar decay pathways. This is consistent with a scenario in which relaxation from S_1_ is dominated by radiative and nonradiative processes from S_1_ to S_0_ with a minor contribution from a decay path involving intersystem crossing to the triplet manifold and leading to the long-lived component. As such this suggests, again similar to M4AC, that the ^1^nπ* state lies too high in energy to efficiently participate in internal conversion and subsequent intersystem crossing. However, although for vertical excitation ^1^nπ* state is indeed S_3_ (see Table 1), our calculations do not provide reliable adiabatic excitation energies for the higher-lying singlet states and therefore do not allow for further validation of this interpretation.

Figure 5d reports time-resolved (1 + 1′) PE spectra of M2AC obtained at 27475 cm^–1^. From the onset of highest-energy band at 1.8 eV we determine a value of 2.3 eV for the adiabatic S_0_ → D_0_ ionization energy. In view of the large geometry changes that occur upon excitation to S_1_ that might moreover involve tunneling in a double well potential, we have indicated as a guide in Figure 5d for the ionization pathway to D_1_ the photoelectron kinetic energy predicted for vertical S_0_ → D_1_ ionization. As we find that geometry optimization of the T_1_ state always leads to a perpendicular geometry of the C_7_=C_8_ bond, we have indicated in this Figure the photoelectron kinetic energy predicted on the basis of the vertical S_0_ → T_1_ excitation energy. Irrespective of these predictions, the similarities between the spectra of M2AC and those of M3AC and M4AC and the previously made assignments for the latter two make us confident that the band around 1.8 eV should be assigned as associated with the S_1_ → D_0_ ionization pathway, and the band around 0.8 eV to S_1_ → D_1_ ionization. Importantly, and in agreement with the previously discussed pump–probe experiments using ion detection, for longer delays a small band around 0.4 eV remains that is assigned to ionization of the T_1_ state of M2AC to D_0_. These results show that the decay dynamics of M2AC are nearly identical to that of M4AC, despite their large differences in vibrational activity and excited state geometry.

### Methyl 4-Dimethylaminocinnamate

In view of the changes in the spectroscopic and dynamic properties of the cinnamate scaffold that have now been observed using an amino instead of a methoxy of hydroxy substituent -and especially the large reduction of the triplet-mediated decay pathway in M4AC- it is of interest to investigate a *para* substitution with a dimethylamino group, as it is a stronger electron-donating group than the amino substituent. Figure 6a shows to this purpose the (1 + 1′) R2PI excitation spectrum of methyl 4-dimethylaminocinnamate (MeM4AC). Similar to M4AC, the excitation energy of S_1_ was found to be larger than half of the adiabatic S_0_ → D_0_ ionization energy allowing for an accurate determination of this quantity as will be shown later. Interestingly, we also found that the excitation laser power had to be reduced significantly to about 10 μJ/pulse to prevent saturation of the S_0_ → S_1_ transition and reduce the contribution of one-color (1 + 1) ionization. We thus infer that the oscillator strength of the S_0_ → S_1_ transition increases by using a stronger electron donating substituent as is also supported by our calculations (Table 1). Based on the previously discussed results for M4AC and the computed optimized geometries of MeM4AC, we conclude that MeM4AC is likely present in two conformations, s-*cis* and s-*trans* conformations but no further UV–UV depletion experiments have been performed to confirm this experimentally.

**Figure 6:**
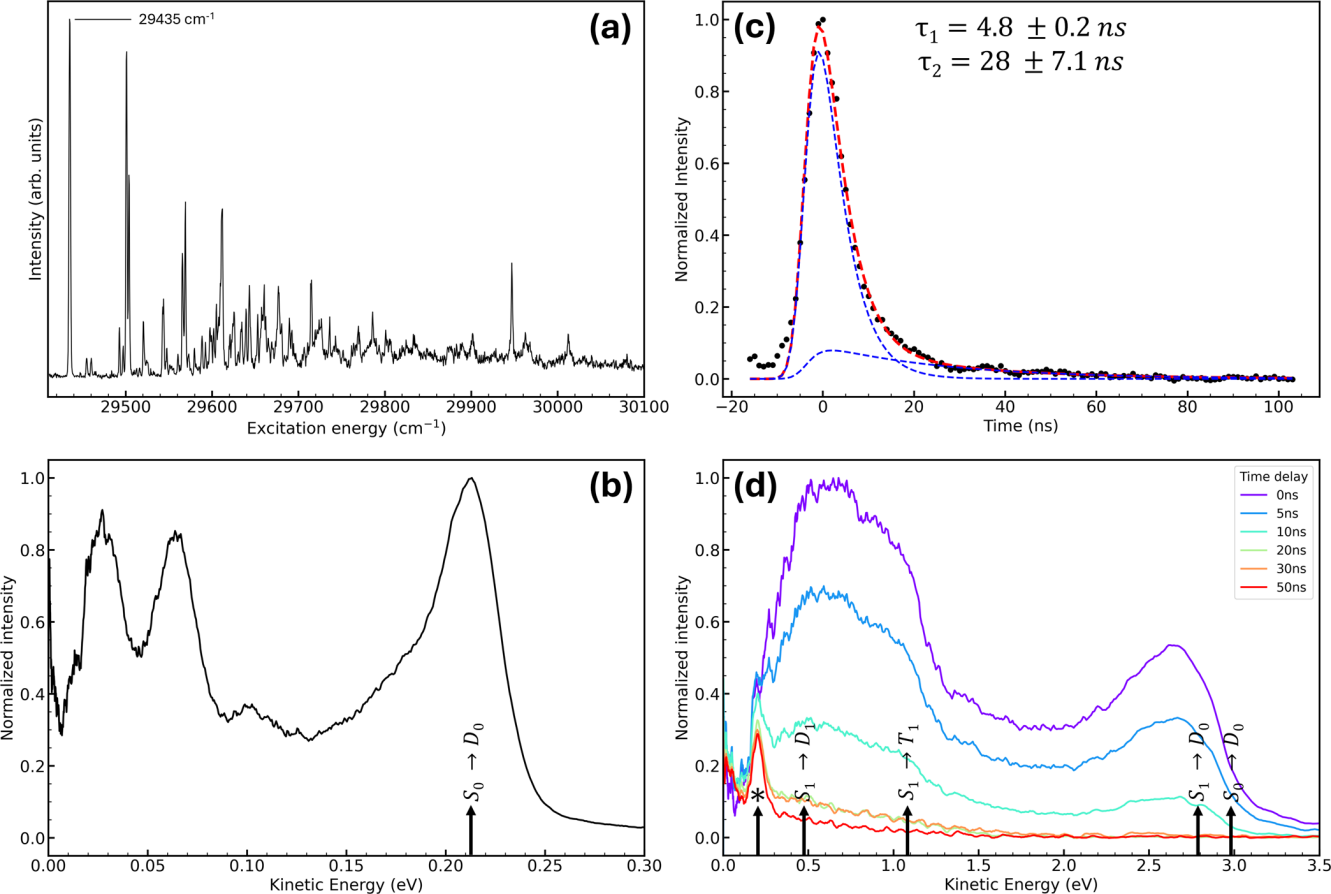
(a) (1 + 1′) R2PI excitation spectrum of MeM4AC. (b) Pump–probe ion yield of MeM4AC probed at 29435 cm^–1^. The black dots represent the data, the red dashed line is the full fit of the data, while the blue dashed lines depict the individual exponential decay components. (c) (1 + 1) PE spectrum of MeM4AC probed at 29435 cm^–1^. (d) (1 + 1′) PE spectra of MeM4AC excited at 29435 cm^–1^ and ionized at 193 nm recorded at different time delays between excitation and ionization laser pulses. The transition annotated by an * correspond to the remaining (1 + 1) signal that could not completely be suppressed.

The R2PI excitation spectrum of MeM4AC is red-shifted with respect to M4AC, the lowest-energy transition at 29435 cm^–1^ lying 1263 cm^–1^ below that of M4AC. This observation further supports a reduction of the HOMO–LUMO gap induced by the strong electron-donating character of the dimethylamino group. Similarly to M4AC, a pronounced in-plane C_4_–C_7_=C_8_ bending mode *b* at 66 cm^–1^ is observed in the excitation spectrum of MeM4AC. The Franck–Condon activity in this spectrum is relatively limited, although somewhat more pronounced than in M4AC, suggesting that the S_1_ excited state does not undergo significant displacements along out-of-plane coordinates.

Figure 6b shows the time-resolved ion yield of MeM4AC probed at 29435 cm^–1^. Convolution of a biexponential decay with the instrumental response function leads to decay times of 4.8 ± 0.2 ns and 28 ± 7.1 ns, respectively, contributing approximately 95.0% and 5.0% to the total signal. These two decay times are assigned in the same manner as for M4AC, that is, the faster one we associate with the decay of S_1_ and the slower one with decay of T_1_. We find that pump–probe measurements performed at higher excitation energies are also well described by a biexponential model but notice that the relative amplitude of the long-lived component increases gradually with excitation energy. It would thus appear that the intersystem crossing quantum yield is somewhat energy dependent. The fitted lifetimes and their relative amplitudes in MeM4AC are essentially identical to those observed for M4AC, indicating very similar excited-state decay dynamics. Thus, the stronger electron-donating character of the dimethylamino group does not appear to alter the decay dynamics observed in M4AC significantly.

One-color (1 + 1) R2PI at 29435 cm^–1^ gives rise to the PE spectrum depicted in Figure 6c. At this excitation energy absorption of two photons brings the molecules just above the D_0_ adiabatic ionization energy, allowing for partial vibrational resolution to be retained in the VMI image. From the PE spectrum we determine the adiabatic ionization energy of MeM4AC as 7.087 ± 0.005 eV. Compared to the ionization energy determined for the s-*cis* conformer of M4AC (7.444 ± 0.005 eV), substitution of the amino group by a dimethylamino group thus lowers the ionization energy by 0.357 eV, indicating that the HOMO is further destabilized as would indeed be expected on the basis of the stronger electron-donating character of the dimethylamino group. Similar to M4AC, the main band in the one-color photoelectron spectrum of MeM4AC at 0.212 eV displays unresolved low-frequency vibrational activity that most like consists of a progression involving the in-plane C_4_–C_7_=C_8_ bending mode of the ion. It is interesting to see that in the higher-frequency region between 800 and 1600 cm^–1^ significantly more vibrational activity is observed than for M4AC, indicating that for MeM4AC larger structural changes occur upon ionization from S_1_ than for M4AC.

Further insight into the electronic and dynamic properties of the S_1_ state of MeM4AC has been obtained from two-color PE spectra of MeM4AC recorded following excitation at 29435 cm^–1^ and ionization at different pump–probe delays (Figure 6d). For zero delay, two main bands are observed. The high kinetic energy band at 2.8 eV is assigned to electrons originating from the S_1_ → D_0_ ionization pathway for which a kinetic energy of 2.8 eV is predicted. The band around 0.8 eV, on the other hand, contains contributions from both the S_1_ → D_1_ and T_1_ → D_0_ ionization channels, for which photoelectron kinetic energies are predicted of 0.5 and 1.1 eV, respectively. At longer time delays, both bands decay rapidly, indicating that these photoelectrons predominantly originate from the initially populated S_1_ state. After a delay of 20 ns, only a weak structure around 0.5 eV remains, which is attributed to electrons produced through the T_1_ → D_0_ ionization pathway. The sharp feature observed near 0.2 eV at these longer delays corresponds to a residual signal from one-color (1 + 1) ionization at 29435 cm^–1^ that could not be completely suppressed (see Figure 6d). This transition is shown on the spectra with an arrow annotated by an *. Nevertheless, the two-color photoelectron spectrum of MeM4AC and its time dependence is nearly identical to that of M4AC, indicating very similar excited-state decay dynamics. We therefore conclude that the S_1_ state predominantly decays through radiative and nonradiative pathways back to the ground state, while a much smaller part of the population relaxes back via a triplet-mediated pathway as was also concluded from the time-dependent pump–probe ion-yield measurements on MeM4AC.

## Conclusions

We have performed a comprehensive study of the spectroscopy and dynamics of electronically excited states of M4AC, M3AC, M2AC and MeM4AC using R2PI, UV–UV depletion, pump–probe ion yield, and photoelectron velocity map imaging spectroscopy. These studies show that the strong electron donating character of the NH_2_ and NMe_2_ groups significantly reduces the excitation energy of the S_1_(^1^ππ*) state, thereby leading to a modification of the excited-state ordering compared to previously studied cinnamate derivatives bearing oxygen-based moieties. An important consequence of this reordering is that for the *para*- and *ortho*-substituted compounds the relaxation cascade to a lower-lying ^1^nπ* state followed by intersystem crossing to a long-lived ^3^ππ* state is to a large extent suppressed, in this way suppressing potential detrimental side reactions. For the *meta* substituted compound, the ^1^nπ* state appears to be energetically inaccessible, resulting in relaxation occurring solely from the S_1_ state. Time-resolved pump–probe measurements are consistent with this picture. M3AC displays a monoexponential decay dominated by radiative decay to the ground state, while M4AC, M2AC and MeM4AC exhibit biexponential decay behavior dominated by a 5–10 ns component attributed to direct relaxation from the S_1_ state and a weak long-lived component to a minor triplet-mediated pathway. Such a conclusion finds further support in the time dependence of photoelectron spectra in which bands can clearly be attributed as being associated with ionization of the S_1_ or T_1_ state.

The vibrationally resolved excitation spectra of M4AC, M3AC and MeM4AC indicate that these compounds retain a largely planar geometry in their first excited state, consistent with a modest structural reorganization upon excitation. M2AC, in contrast, exhibits dominant vibrational activities, pointing to large geometric changes in the excited state that likely arise from interactions between the *ortho*-amino group with the methyl-acrylate tail of the cinnamate. Previously we have observed that steric hindrance on the C_7_=C_8_ bond disrupting conjugation of the acrylate double bond to the phenyl part has a major impact on the excited-state relaxation dynamics.^24^ It is interesting to notice that for a case where steric interactions might similarly affect the conjugation between the phenyl and acrylate parts apparently leaves the excited-state dynamics relatively unaffected.

Together, these results demonstrate that electron-donating amino substituents provide an effective means of controlling the excited-state ordering and relaxation pathways of cinnamate-based chromophores. In particular, amino substitution strongly reduces the population of long-lived triplet states, which are often associated with undesirable photochemical side reactions. These findings therefore establish a promising molecular design strategy for the development of safer and more photostable UV-absorbing materials. Future work will focus on extending these studies to solvated environments in order to determine to what extent the excited-state relaxation mechanisms identified in the gas phase are modified under conditions in which such UV filters would be applied.

## Supplementary Material



## Data Availability

The data supporting the findings of this study are available from the corresponding authors upon reasonable request.

## Footnotes

1 Calculated for M4AC using the Beyer–Swinehart algorithm^41^

## Notes

Table S1 with relative room-temperature Gibbs energies of conformers of M3AC and their relative predicted S_0_ → S_1_ 0–0 transition energies (PDF)
