# Supplementary material for: Jet-Cooled Spectroscopy of Amino-Substituted Cinnamates
Source: J Phys Chem A. 2026 Aug 1;130(32):6409–21. doi: 10.1021/acs.jpca.6c04208 (PMC13479610; doi:10.1021/acs.jpca.6c04208)
Supplement: Supplementary file 1 [file jp6c04208_si_001.pdf]

Supporting Information

Jet-Cooled Spectroscopy of Amino-Substituted Cinnamates

Hugo Maurer[†] and Wybren Jan Buma[*,†,‡]

[†] *Van't Hoff Institute for Molecular Sciences, University of Amsterdam, Science Park 904, 1098 XH Amsterdam, The Netherlands*

[‡] *Radboud University, Institute for Molecules and Materials, FELIX Laboratory, Toernooiveld 7c, 6525 ED Nijmegen, The Netherlands*

Table S1. Relative ωB97XD/cc-pVDZ room-temperature Gibbs energies of conformers of M3AC and their relative predicted $S_0 \rightarrow S_1$ 0-0 transition energies.

**Conformer**	**Gibbs energy (kcal/mol)**	**$S_0 \rightarrow S_1$ 0-0 transition (cm^{-1})**
anti/s-cis	0.00	0
syn/s-cis	0.00	49
syn/s-trans	0.59	52
anti/s-trans	0.97	146
